# Comparative Evaluation of Anthelmintic Activity of Edible and Ornamental Pomegranate Ethanolic Extracts against* Schistosoma mansoni*

**DOI:** 10.1155/2016/2872708

**Published:** 2016-11-21

**Authors:** Doaa A. Yones, Dalia M. Badary, Hesham M. B. Sayed, Soad A. H. Bayoumi, Azza A. Khalifa, Ahmed M. El-Moghazy

**Affiliations:** ^1^Parasitology Department, Faculty of Medicine, Assiut University, Assiut 71526, Egypt; ^2^Pathology Department, Faculty of Medicine, Assiut University, Assiut 71526, Egypt; ^3^Pharmacognosy Department, Faculty of Pharmacy, Assiut University, Assiut 71526, Egypt

## Abstract

Due to the development of praziquantel (PZQ) schistosomes resistant strains, the discovery of new antischistosomal agents is of high priority in research. This work reported the* in vitro *and* in vivo* effects of the edible and ornamental pomegranate extracts against* Schistosoma mansoni*. Leaves and stem bark ethanolic extracts of both dried pomegranates were prepared at 100, 300, and 500 *μ*g/mL for* in vitro* and 600 and 800 mg/kg for* in vivo*. Adult worms* Schistosoma mansoni* in RPMI-1640 medium for* in vitro* and* S. mansoni *infected mice for* in vivo* tests were obtained from Theodor Bilharz Research Institute, Cairo, Egypt.* In vitro* activity was manifested by significant coupled worms separation, reduction of motor activity, lethality, and ultrastructural tegumental alterations in adult worms.* In vivo* activity was manifested revealed by significant reduction of hepatic granulomas number and diameter, decreased number of bilharzial eggs in liver tissues, lowered liver inflammatory infiltration, decreased hepatic fibrosis, and inducible nitric oxide synthase (iNOS) expression. Ethanolic stem bark extract of edible pomegranate exhibited highest antischistosomal activities both* in vitro* and* in vivo*. Therefore, pomegranate showed a good potential to be used as a promising new candidate for the development of new schistosomicidal agents.

## 1. Introduction

Schistosomiasis is one of the world major public health problems, caused by the blood-dwelling fluke of the genus* Schistosoma*. The clinically worldwide relevant species are* S. mansoni*,* S. haematobium*, and* S. japonicum* [1]. In addition to the previously mentioned species,* S. mekongi* and S.* intercalatum* represent the most important pathogenic species for human beings. Depending on the species, the schistosome worms persist in the liver and hepatic portal system or the urinary tract system of humans. Mature schistosomes lay eggs within their host, which often get trapped in the host's tissues, resulting in inflammatory and obstructive diseases of the affected organs [2, 3].

Schistosomiasis is one of the most widely occurring neglected tropical diseases with high levels of incidence in Asia, Africa, and Latin America. Studies have shown approximately 779 million people living at higher risk of infection, and 239 million people are infected with schistosomes [4, 5].

To cure morbidity and prevent the development of severe chronic stage hepatosplenomegaly, praziquantel (PZQ) is the only choice of chemotherapy against all species of* Schistosoma*. Presenting good efficacy and low toxicity, the drug has been widely used for more than three decades and is therefore susceptible to the emergence of praziquantel resistant schistosomes [6].

The study of medicinal plants as a new approach for schistosomiasis treatment is feasible and promising one [7, 8]. The research on medicinal plants is encouraged by the WHO, considering that certain traditional knowledge on curative plants could add up to the development of new pharmaceutical products as well as to the combat against diseases that affects the populations of developing countries [9, 10].

The search for new antischistosomal treatments has led to the study of natural substances such as artemisinin and its derivatives, curcumin, phytol, and pomegranate [11–13].

Pomegranate (*Punica granatum* L.) is a favorite table fruit in tropical countries, belonging to Punicaceae family. This family is unusual in having the sole genus* Punica, *a genus of large shrubs or small trees with two species. One is* P. protopunica* Balf. S., which is found wild in Socotra island, and the other is* P. granatum *L. (edible pomegranate), which is cultivated in tropical and subtropical parts of the world for its edible fruits [14].

*Punica granatum* L. var.* nana* is a dwarf variety of* P. granatum* L. popularly planted as an ornamental plant in gardens. It could well be a wild form with distinct origin. It does not usually produce edible fruits [15].

The peel and seeds of* P. granatum *L. showed various therapeutic applications such as antibacterial, antifungal, antioxidant, antitumor, antiviral, antimalarial, and antimutagenic effects [16, 17]. Edible pomegranate extracts have been reported to have promising results against* S. mansoni* either* in vitro* or* in vivo* [12, 18, 19].

The root, stem bark, and, to lesser extent, the fruit rind of pomegranate have been commonly used as vermifugal or taenicidal agents [20]. Pomegranate has also antiprotozoal activity and it is used in folk medicine for treatment of dysentery [21].

The methanolic extract of* P. granatum* L. var.* nana* leaves exhibited antioxidant activity, nematicidal activity against three root-knot nematode species, and hepatoprotective activity against carbon tetrachloride induced hepatotoxicity [22].

In our locality, many factors, including the high prevalence, wide distribution of schistosomiasis, and drug resistance for the already used treatment, necessitate the need for control of this helminthiasis and impulse the studies for new and more comprehensive alternative therapeutics without adverse effects. Hence, the current study aimed to investigate the* in vitro* and* in vivo* activity of leaves and stem bark ethanolic extracts of both dried edible and ornamental pomegranate against* Schistosoma mansoni* (Egyptian strain).

## 2. Materials and Methods

### 2.1. Plant Materials

200 g of each of the leaves of edible pomegranate (LEP) and stem bark of edible pomegranate (SEP) (*P. granatum* L.) and similar leaves of ornamental pomegranate (LOP) and stem bark of ornamental pomegranate (SOP) of* P. granatum* L. var.* nana* was locally collected from Faculty of Pharmacy garden of medicinal plants and Experimental Station of Agriculture, Faculty of Agriculture Assiut University, Assiut, Egypt. Test plants were authenticated by Dr. Naeem E. Keltawy, Professor of Ornamental Horiculture and Floriculture, Faculty of Agriculture, Assiut University, Assiut, Egypt. Voucher specimens (number 45) were kept in the herbarium, Pharmacognosy Department, Faculty of Pharmacy, Assiut University.

### 2.2. Preparation of Extracts

Plant samples were allowed to dry at room temperature before being ground to fine powder. Powdered plant materials were extracted with 70% ethanol at room temperature by maceration and then filtered and the filtrates were concentrated under vacuum using rotary evaporator. The obtained solvent-free residue of each plant extract was stored at 4°C for subsequent preparation of the required doses.

### 2.3. Dose Preparations of Extracts

Plant extracts were freshly prepared before usage by suspending 1 g of each extract in 50 mL 3% tween 80 dissolved in 0.9% saline. For* in vitro* antischistosomal testing, 100, 300, and 500 *μ*g/mL were used, while 600 and 800 mg/kg for* in vivo* assay were prepared [12].

### 2.4. Cytotoxicity Assays (CTAs) on Tissue Culture Cells

CTAs were performed on mouse fibroblast cell BALB/c 3T3 (VACSERA, Egypt) supplemented with 10% bovine calf serum, 4 mM L-glutamine, 100 IU penicillin, and 100 *μ*g/mL streptomycin (Bioanalyse, Turkey) using the neutral red uptake assay for all pomegranate extracts at the higher concentration [23].

### 2.5. Evaluation of Microbial Contamination and Endotoxin Production

Total aerobic microbial count and total combined yeasts/moulds count were used for quantitative enumeration of mesophilic bacteria or fungi that may grow under aerobic conditions in all pomegranate extracts at the higher concentration and for PZQ using the pour plating technique (EDQM Council of Europe, 2014). The bacterial endotoxin test was performed by the limulus amoebocyte lysate assay (gel-clot technique) as reported by Hussaini and Hassanali [24].

### 2.6. Standard Antischistosomal Treatment

Praziquantel suspension, a product of Egyptian International Pharmaceutical Industries Company (EIPI Co.), was purchased locally. PZQ was used as positive control at concentration of 10 *μ*g/mL for* in vitro* and 200 mg/kg for* in vivo* experiments [25, 26].

### 2.7. Schistosome Parasites and Experimental Infected Hosts

*S. mansoni* (Egyptian strain) adults were purchased from the experimental animal research unit of the Schistosome Biological Supply Center (SBSC), Theodor Bilharz Research Institute (TBRI), Cairo, Egypt. Swiss albino female mice CD strain, weighing 20–25 g and aged 4 weeks, were individually infected using the tail immersion technique by exposure to a suspension containing 100* S. mansoni *cercariae (100 cercariae/mouse) from naturally infected* Biomphalaria alexandrina* for 2 h according to the method described by Liang et al. [27]. Mice were bred under environmentally controlled conditions (temperature ~25°C and 12 h light and dark cycle) and fed with a standard stock commercial pellet diet (containing 24% protein) and water* ad libitum*.

## 3. Experimental Design

### 3.1. Experiment (1)

#### 3.1.1. *In Vitro* Assessment of the Antischistosomal Effects of the Prepared Extracts

For* in vitro* bioassay,* Schistosoma mansoni* adult worm pairs of Egyptian strain were retrieved aseptically from sacrificed infected mice and collected by perfusion of the hepatic portal system and mesenteric veins using citrated saline according to the technique of Stirewalt and Dorsey [28] from mice livers 8 weeks postinfection.

Adult worms were washed three times with the RPMI 1640 (Roswell Park Memorial Institute 1640) culture medium (Invitrogen, Carlsbad, California, USA), which was used for culturing the parasite. The medium was supplemented with L-glutamine, 20% fetal calf serum, and antibiotics (300 *μ*g streptomycin, 300 IU penicillin, and 160 *μ*g gentamycin per mL) [12]. After washing, 5 couples of worms were transferred to each well of a 24-well culture plate (TPP, St. Louis, MO) containing the same medium.

Two mL of the tested doses (100, 300, and 500 *μ*g/mL) from leaves and stem bark extracts was added to each well. The final volume in each well was 2 mL. The plate was incubated at 37°C in a humid atmosphere containing 5% CO_2_ [29]. The parasites were kept for 12 h and monitored every 2 h. A pure medium and medium with 3% tween 80 in 0.9% saline were used as negative controls, while PZQ (10 *μ*g/mL) was used as a positive control. All the steps were performed under a sterilized laminar flow chamber. The experiment was carried out in triplicate and repeated three times.

Treated worms were monitored for their mating (pairing) of the worms, motility (worm's motor activity changes), and mortality rate using an inverted optical microscope (Olympus CK2). Worms which did not show motility for one minute were considered dead. Changes in worm's motor activity (motility) of schistosomes were assessed qualitatively and their motor activity reduction was defined as “slight” or “significant” [30].

The effect of the treatment was also assessed with an emphasis on morphological alterations in the tegument which were observed using scanning electron microscopy (SEM) [31]. Observation of adult schistosomes in the* in vitro* experiment was performed at 2 h intervals throughout the 12 h experimental incubation period and the results were reported at 2, 4, 6, and 12 h (the end point of the experiment for the negative control groups).

#### 3.1.2. Preparation of Adult* S. mansoni* Worms for SEM

To observe morphological changes in the tegument of adult parasites, schistosome worms, when they died, and control worms at 12 h, the end point of the experiments, were washed thoroughly with distilled water. The parasites were fixed for 2 h in 4% glutaraldehyde (pH 7.4) and 5% paraformaldehyde in 0.1 M cacodylate buffer (pH 7.2). They were rinsed overnight in cacodylate buffer, dehydrated, dried in a critical point dryer according to Hayat [32], mounted on stubs, and sputter-coated with gold particles in the sputter coating apparatus for 6 minutes. Specimens were processed, examined, and photographed using Jeol-JSM-5400 LV at the Scanning Electron Microscope Unit, Assiut University, Assiut, Egypt.

### 3.2. Experiment (2)

#### 3.2.1. *In Vivo* Assessment of the Antischistosomal Effects of the Prepared Extracts

Fifty-five* S. mansoni* infected female mice were obtained from TBRI, Cairo, Egypt, 8 weeks postinfection. Infected mice were randomly allocated into 11 groups with 5 animals each, at the time of the experiment:

Infected untreated control mice (negative control 1)
Infected mice given 3% tween 80 in saline (negative control 2)
Infected mice treated with 200 mg/kg PZQ (positive control)
Infected mice treated with 600 mg/kg LEP
Infected mice treated with 600 mg/kg SEP
Infected mice treated with 800 mg/kg LEP
Infected mice treated with 800 mg/kg SEP
Infected mice treated with 600 mg/kg LOP
Infected mice treated with 600 mg/kg SOP
Infected mice treated with 800 mg/kg LOP
Infected mice treated with 800 mg/kg SOP

Each mouse was given a single oral dose daily for 7 consecutive days using stainless-steel esophageal tube. All mice were sacrificed by cervical dislocation after 7 days of treatment. Assessment of the treatment* in vivo* was performed through histopathological examination of liver tissue for detection of hepatic inflammation, hepatic fibrosis, and schistosomal granulomas formation. The assessment was also done through immunohistochemical analysis of iNOS reactivity in liver tissue. The experiment was repeated three times.

#### 3.2.2. Histopathological Assessment

Liver samples of the left lobe of each sacrificed mouse were rinsed with phosphate-buffered saline and fixed in 10% formalin for 24 h. Liver samples were dehydrated in increasing concentrations of ethanol, diaphonized in xylol, and embedded in paraffin wax blocks. Sections of 4 *μ*m thickness were stained with hematoxylin and eosin [33]. The sections were evaluated using the bright field microscopy to evaluate the degree of inflammation, fibrosis, and granuloma formation followed by image capture and processing using Camidia image manager. All the granulomas found in 10 histologic sections of random fields were counted. Measurement of mean granuloma diameter was performed using an ocular micrometer at magnification of 100x. Only nonconfluent, lobular granulomas containing eggs in their centers were measured (periocular granulomas) [34].

#### 3.2.3. Immunohistochemistry for Determination of iNOS Reactivity

Left lobe liver sections from the previously prepared paraffin blocks, 4 *μ*m thickness, mounted on glass slides, were kept overnight at 56°C. They were deparaffinized with xylene and rehydrated with decreasing percentages of ethanol and finally with water. For antigen retrieval, slides were heated by microwaving in 10 mM citrate buffer (pH 6.0) for 12 min. Slides were left to cool for 20 min at room temperature and rinsed with distilled water. Surroundings of the sections were marked with a PAP pen. The endogenous peroxidase activity was blocked with H_2_O_2_ for 10 min at room temperature and later rinsed with distilled water and PBS (phosphate- buffered saline). Liver sections were then incubated for 1 h at room temperature with the following antibody: iNOS rabbit Pab (Neomarker, RB-1605-P) antibodies. Antibodies were diluted at 1 : 100. The sections were washed and rinsed with PBS three times for 5 min each. Slides were incubated for 30 min at room temperature with biotinylated goat anti-rabbit antibodies. The streptavidin peroxidase label reagent was applied to the slides after being washed in PBS, for 30 min at room temperature in a humid chamber. After blotting off excess buffer, a universal staining colored product was developed by incubation with AEC (3-Amino-9-Ethylcarbazole) Chromogen (Lab Vision, TA-004-HAC) for 5 min according to manufacturer's instructions. Finally, slides were dehydrated and cleared. The slides were counterstained with Mayer's hematoxylin and mounted in glycerol gelatin after washing in distilled water and mounted with cover slips [35].

### 3.3. Ethical Considerations

A standard protocol was drawn up in accordance with the Good Laboratory Practice (GLP) regulations of the World Health Organization (WHO). The principles of laboratory animal care were duly followed in this study [36]. Ethical animal practices were followed under standard regulations dictated by the animal care committee of Faculty of Medicine, Assiut University. Ethical approval was granted by the Research and Ethic Committee of Faculty of Medicine, Assiut University.

### 3.4. Statistical Data Analysis

The results were analyzed using the SPSS (Statistical Package for the Social Sciences, version 16 for Windows) software (SPSS Inc., Chicago, Illinois, USA). Significant differences were determined by one-way analysis of variance (ANOVA). The values were presented as mean ± standard deviation (SD). Data were analyzed using Student's Tukey's test (*t*-test) which was used to calculate the significance of differences observed between mean values of experimental and control groups in each experiment. *P* values of less than 0.05, 0.01, or 0.001 were used to indicate statistical significance.

## 4. Results

The preliminary phytochemical screening of both plant extracts showed the presence of volatile constituents, polyphenols glycosides, triterpenes, sterols, flavonoids, anthocyanins, triglycerides, tannins, and alkaloids.

### 4.1. Cytotoxicity Assays (CTAs)

The optical density (OD)_540_ of each of the tested extracts was compared with the mean value OD_540_ for the negative control (distilled water). Tested pomegranate extracts fulfilled the mentioned acceptance criteria through absence of cytotoxic effects of the studied extracts. Cell viabilities were more than 70% relative to the negative control for tested extracts at their highest concentrations. Thus, the concentrations in which pomegranate presented the schistosomicidal activity were not associated with cytotoxic effects on fibroblast cell.

### 4.2. Evaluation of Microbial Contamination and Endotoxin Production

Total aerobic microbial count and total combined yeasts/moulds count were negative for the tested pomegranate extracts. Tested pomegranate extracts were endotoxin free.

### 4.3. *In Vitro* Treatment Efficacy of LEP, SEP, LOP, and SOP Extracts on Adults* S. mansoni* at Different Concentrations

All the tested extracts influenced the process of natural mating, causing separation of couple schistosomes depending on the concentration used and exposure time. Nearly 95% of the worms had been separated within the first 2 h with the use of 500 *μ*g/mL SEP, compared to the negative control groups (Table 1). PZQ (10 *μ*g/mL) caused couple worm separation after the first 2 h of incubation. Negative control groups showed couple separation nearly at 10 h after incubation. Moreover, concentrations which were not 100% lethal to the worms were proven as efficient mating inhibitors, once all of them separated the couples in all samples.

Concerning the motility, a significant reduction in the parasites movements was observed in all concentrations. The percentage of worms that had their motility reduced was directly proportional to the concentration and to the period of incubation. A slight decrease in motor activity was observed after 2 h of incubation for all adult worms exposed to 500, 300, and 100 *μ*g/mL concentration of SEP. Total motility loss occurred at 4, 6, and 12 h, respectively. No change in motor activity was observed at 4 h interval, while it decreased at 6 h interval and complete loss of motility occurred at 10 h interval in the negative control groups. On the other hand, PZQ (10 *μ*g/mL) resulted in decrease in motor activity starting from the first 2 h of incubation and complete loss of motor activity in all worms occurred at 4 h interval.

The survival of* S. mansoni* adults exposed to ethanolic extracts of LEP, SEP, LOP, and SOP depended directly on both concentration and incubation period. The 500, 300, and 100 *μ*g/mL concentrations of SEP caused death of 100% of parasites within 4, 6, and 12 h of incubation, respectively (Table 1). Ethanolic extracts of LEP at 500, 300, and 100 *μ*g/mL concentrations caused death of 100% worms after 6 and 12 h of incubation, respectively (Table 1). LOP and SOP (500 *μ*g/mL) caused significant mortality (*P* < 0.01) among schistosome parasites after 6 h of incubation, while 100 and 300 *μ*g/mL concentrations of the same extracts expressed their mortality effect on adults* S. mansoni* after 12 h of incubation (Table 1). No difference was observed between male and female adult worms in response to different concentrations of the used extracts in either motility affection or survival rates.

The PZQ treated group (positive control) showed total death of the parasites (100%) after 4 h of incubation (Table 1). All the negative control groups were killed at 12 h of incubation which was considered the end point of the experiment.

### 4.4. Tegumental Changes of* S. mansoni* Adult Worms in Response to Exposure to Edible and Ornamental Pomegranate Extracts Visualized by Scanning Electron Microscope (SEM)

**Figure 1:**
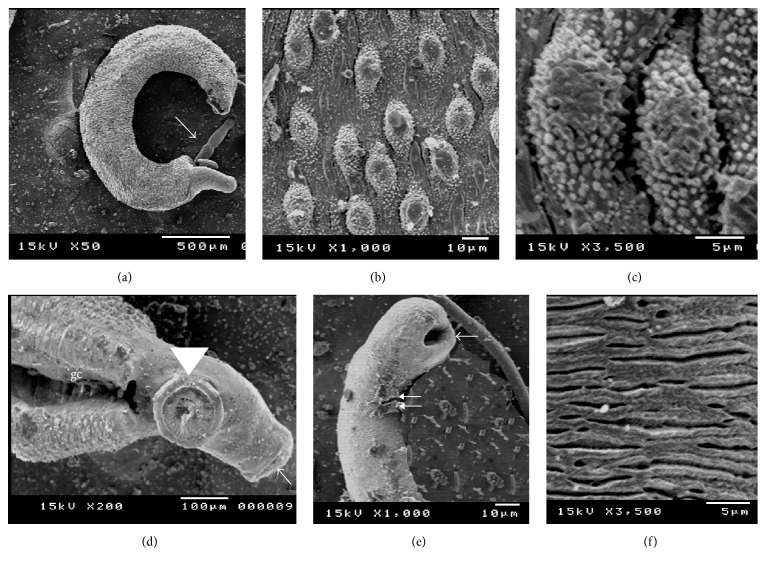
Scanning electron microscopy (SEM) of normal adult* S. mansoni* kept in RPMI-1640 alone or with 3% tween 80; (a) male and female (arrow) in copula; (b) male dorsal surface showing tubercles and spines; (c) higher magnification of the tubercles and spines; (d) male anterior end showing gynecophoric canal (gc), oral sucker (arrow); and ventral sucker (arrowhead); (e) female anterior end showing oral sucker (arrow), and ventral sucker (double arrows); (f) female dorsal surface showing normal appearance of the tegument.

Ultramorphological alterations were observed in* S. mansoni* adult males and females after 12 h incubation* in vitro* with the 100, 300, and 500 *μ*g/mL concentrations of leaves and stem bark of both edible and ornamental pomegranate ethanolic extracts. The parasites exposed to LEP and SEP revealed dose-dependent variable degrees of tegumental morphological alterations, when compared to negative control ones. SEP (500 *μ*g/mL) induced more morphological destructions than those induced by LEP at the same dose. No tegumental changes in adult worms were observed for the negative control groups (Figure 1). The positive control (PZQ treated group) showed similar tegumental alteration in 100% of schistosome worms.

**Figure 2:**
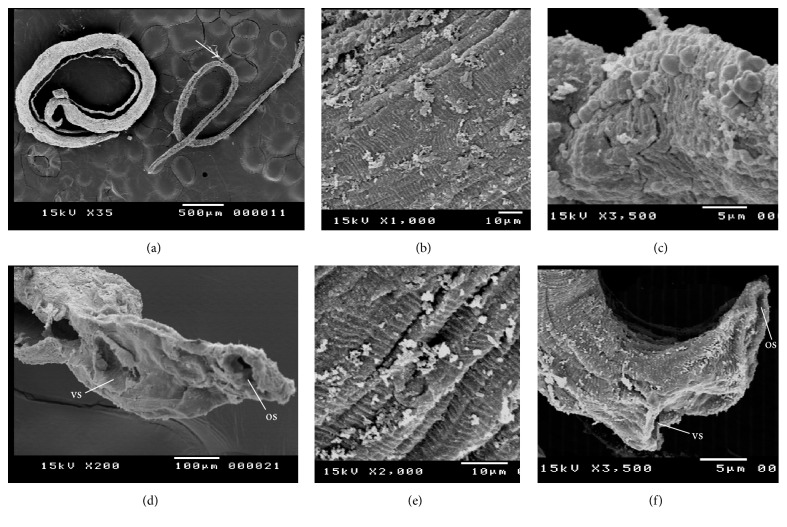
Scanning electron microscopy (SEM) of adult* S. mansoni* worms after their exposure to 100, 300, and 500 *μ*g/mL ethanolic extracts of leaves and stem bark of edible and ornamental pomegranate. (a) Separated male and female (arrow); (b) male dorsal surface showing tegumental peeling with destruction and peeling of tubercles and spines; (c) male showing bubbles surrounding the morphologically altered tubercles on its dorsal surface; (d) male suckers' alterations or destruction, os: oral sucker and vs: ventral sucker; (e) female showing tegumental scaling, wrinkling, and erosion; (f) female suckers' alterations or destruction, os: oral sucker and vs: ventral sucker.

*Male Worms*. The treatments caused variable degrees of tegumental contractions, tubercles, and spine damage (destruction, peeling of spines, tubercles, and tegument peeling or sloughing) especially on its dorsal surface. The occurrence of bubbles surrounding the morphologically altered tubercles was observed in addition to suckers alteration or destruction (Figures 2(a)–2(d)).

*The Female Worms*. Tegument scaling, wrinkling, and erosion (contraction and peeling of dorsal region) and suckers' alterations or destruction were observed (Figures 2(e) and 2(f)).

Concerning treatment with LOP and SOP extracts, the worms (either male or female) showed similar morphological tegumental changes but of lesser degree to LEP and SEP induced morphological changes.

### 4.5. Treatment Efficacy* In Vivo* of SEP, LEP, LOP, and SOP Extracts

#### 4.5.1. Histopathological Assessment

**Figure 3:**
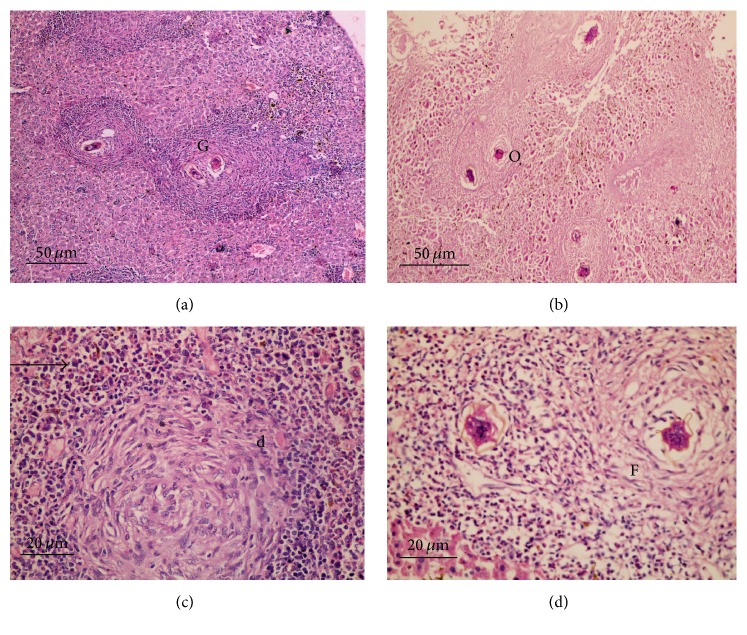
Histological liver sections; H & E staining; (a) infected untreated group showing numerous bilharzial eggs surrounded by numerous chronic inflammatory cells (×200); (b) group treated with 600 mg/kg SOP (×200); (c) group treated with 600 mg/kg LOP (×400); (d) group treated with 600 mg/kg LEP (×400). All showed similar structures: (G) granuloma, (O) bilharzial eggs, and (F) fibrosis and arrow pointed to chronic inflammatory cells.

Microscopical examination of histological liver sections from infected untreated control group revealed pathological chronic granulomatous lesions in the hepatic parenchyma. These lesions formed of numerous bilharzial eggs containing miracidia, surrounded by numerous chronic inflammatory cells in form of epithelioid cells, lymphocytes, plasma cells, macrophages, and eosinophils forming granuloma with severe areas of fibrosis (Figure 3(a)).

**Figure 4:**
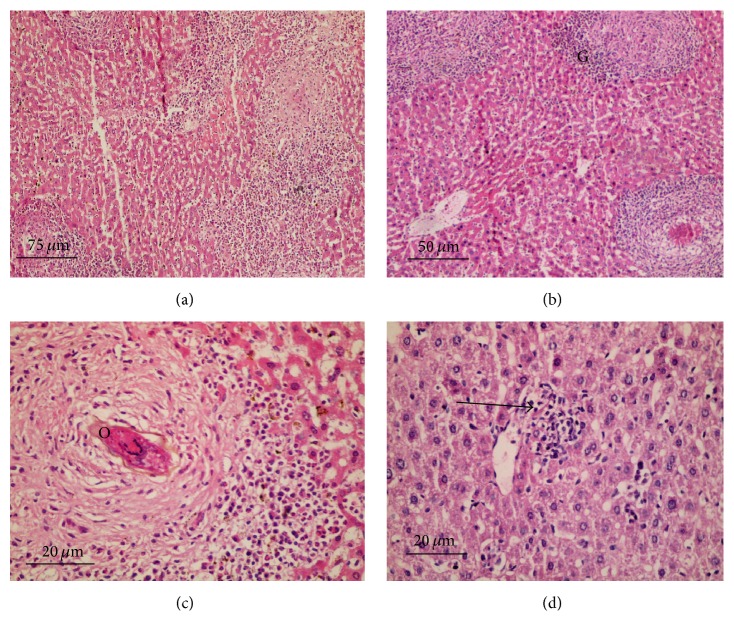
Histological liver sections; H & E staining; (a) group treated with 800 mg/kg LOP (×100); (b) group treated with 800 mg/kg SOP (×200); (c) group treated with 800 mg/kg SOP higher magnification (×400); all showing less prominent inflammatory reactions; (d) group treated with 800 mg/kg LEP showing moderate diffuse infiltration of liver parenchyma by chronic inflammatory cells without observed eggs or areas of fibrosis (×400); (G) granuloma and (O) bilharzial eggs and arrow pointed to chronic inflammatory cells.

Nearly similar observations were detected in the hepatic histological sections from groups treated with 600 mg/kg SOP, LOP, and LEP (Figures 3(b)–3(d)). These inflammatory reactions were less prominent in groups treated with 800 mg/kg LOP and SOP in the form of granuloma with fewer eggs, less fibrosis, and moderate chronic inflammatory cell infiltration (Figures 4(a)–4(c)).

Histological liver sections from groups treated with LEP (800 mg/kg) showed moderate diffuse infiltration of liver parenchyma by chronic inflammatory cells without observed eggs or areas of fibrosis (Figure 4(d)). Similar observations were reported in histological liver sections from groups treated with 600 and 800 mg/kg SEP and PZQ (200 mg/kg). They showed absence of bilharzial eggs and fibrosis with significant reduction of liver parenchyma infiltration by the chronic inflammatory cells. For hepatic granulomas number and diameter, histological liver sections from infected untreated control groups revealed about 121.3 granulomas of average diameter 235.7 ± 16.1 *μ*m. Livers of different treated groups showed decrease in granuloma size and number with minimal degenerative changes in liver tissues as shown in Table 2.

Oral administration of 800 mg/kg LEP decreased hepatic granulomas number and size to 54.1 and 37.2%, respectively, while administration of 600 mg/kg of the same extract decreased them to 51.3 and 34.5%, respectively. Following oral administration of 600 mg/kg SEP, there was decrease in the mean granuloma diameter to 40.1%, while 800 mg/kg of the same extract showed about 40.9% reduction. Other extracts and PZQ treatments showed variable effects on hepatic granuloma number and diameter (Table 2). Thus, the hepatic granuloma average diameter was significantly smaller (*P* < 0.01) in groups treated with SEP and LEP in comparison to groups treated with SOP and LOP (600 and 800 mg/kg) and control groups. These hepatic granuloma average diameters were nearly similar to positive control group (PZQ 200 mg/kg) with insignificant difference (*P* > 0.01) (Table 2).

#### 4.5.2. Expression of iNOS Detected by Immunohistochemistry

**Figure 5:**
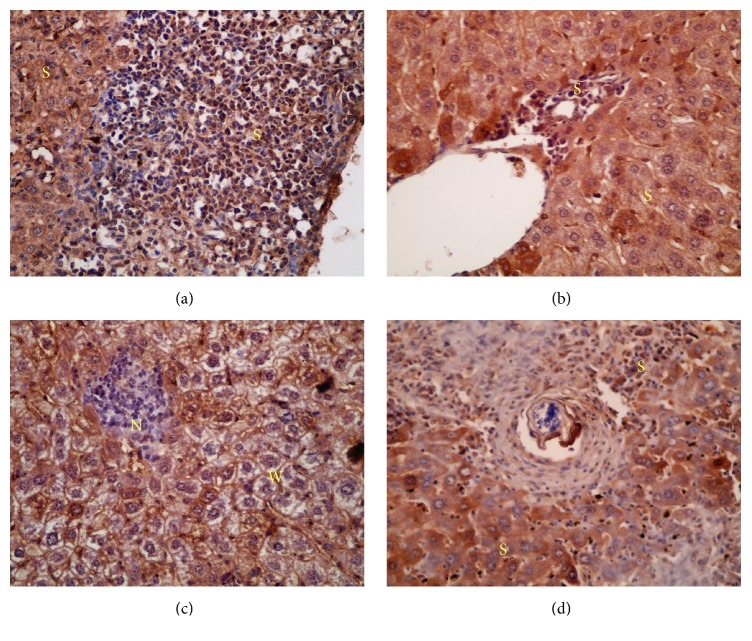
The distribution and intensity of iNOS in liver sections analyzed by immunohistochemistry; (a) infected untreated group; (b) group administered tween 80 showing strong iNOS reactivity (cytoplasmic expression) in the hepatocytes (×400); (c) group treated with PZQ showing lowest iNOS expression (reactivity) in the hepatocytes (×400); (d) groups treated with 600 mg/kg LEP showing similar observation to (a) and (b) (×400); (S) strong, (W) weak, and (N) negative intensity.

**Figure 6:**
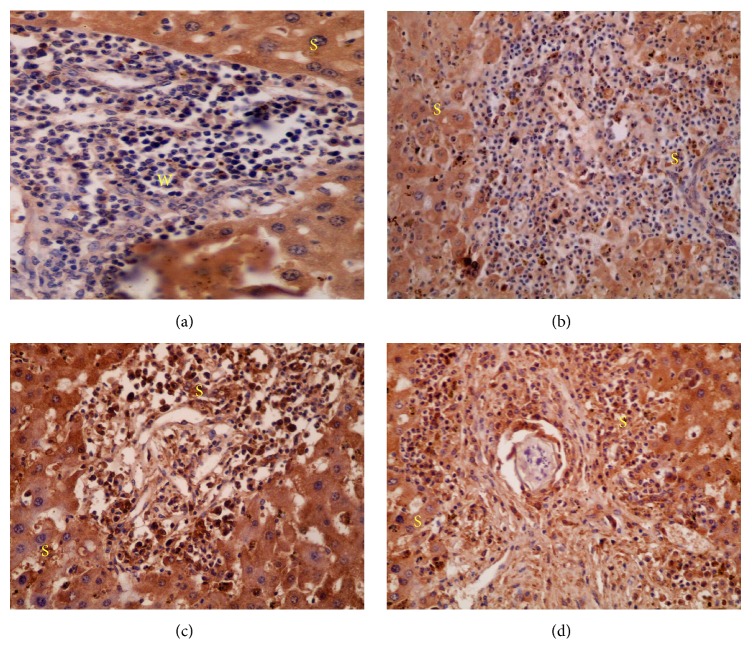
The distribution and intensity of iNOS in liver sections analyzed by immunohistochemistry; (a) group treated with 600 mg/kg SOP (×400); (b) group treated with 600 mg/kg LOP (×200) showing strong iNOS reactivity (cytoplasmic expression) in the hepatocytes; (c) group treated with 800 mg/kg LEP (×200); (d) group treated with 800 mg/kg LOP (×400) showing low or weak expression in the hepatocytes; (S) strong and (W) weak iNOS expression.

**Figure 7:**
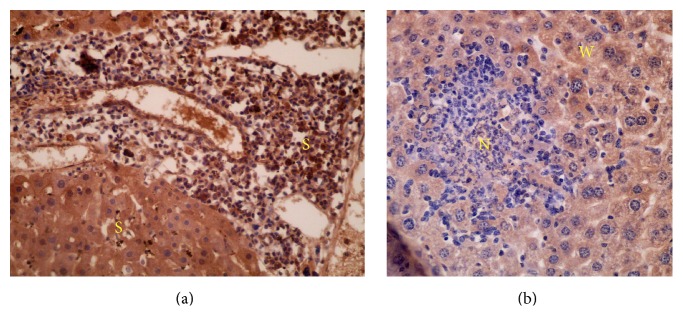
The distribution and intensity of iNOS in liver sections analyzed by immunohistochemistry; (a) group treated with 800 mg/kg SOP showing strong iNOS reactivity (cytoplasmic expression) in the hepatocytes (×200); (b) groups treated with 600 and 800 mg/kg SEP showing lowest iNOS expression (reactivity) in the hepatocytes (×400); (S) strong, (W) weak, and (N) negative iNOS expression.

In comparison to immunohistochemical expression of iNOS between different groups, iNOS reactivity (cytoplasmic expression) was stronger in the hepatocytes of infected untreated group, group administered 3% tween 80 (Figure 5(a)), groups treated with 600 mg/kg LEP (Figure 5(d)), 600 mg/kg SOP (Figure 6(a)), 600 mg/kg LOP (Figure 6(b)), and 800 mg/kg SOP (Figure 7(a)) compared to low or weak expression in the hepatocytes of groups treated with 800 mg/kg LEP (Figure 6(c)) and 800 mg/kg LOP (Figure 6(d)) (Table 2). Groups treated with PZQ (Figure 5(c)) and 600, 800 mg/kg SEP (Figure 7(b)) revealed the lowest iNOS expression (reactivity) in the hepatocytes. The iNOS reactivity was stronger around the granulomatous lesions. Additionally, the iNOS reactivity reduced simultaneously with decreased granulomatous lesions.

Strong reactivity of iNOS was clearly observed in the cytoplasm of the chronic inflammatory cells in infected untreated group (Figure 5(a)), group administered 3% tween 80 (Figure 5(b)), groups treated with 600 mg/kg LEP (Figure 5(d)), 600 mg/kg SOP (Figure 6(c)), 600 mg/kg LOP (Figure 6(b)), 800 mg/kg LOP (Figure 6(d)), and 800 mg/kg SOP (Figure 7(a)), compared to low or weak expression in inflammatory cells of group treated with 800 mg/kg LEP (Figure 6(a)) (Table 2). Negative reactivity of iNOS was observed in the cytoplasm of the chronic inflammatory cells in groups treated with PZQ (Figure 5(c)) and SEP (600, 800 mg/kg) (Figure 7(b)) (Table 2).

## 5. Discussion

Plants are an important source of biologically active compounds that can provide structures for the development of new drugs [37]. In recent years, an extensive attention to natural products as a treatment of neglected tropical diseases, including schistosomiasis, has been growing. The awareness has stimulated an exertion to improve a new medicine as a substitute method to this parasitosis control [9, 10, 38].

In this study, the effects of different concentrations of leaves and stem bark ethanolic extracts of dried edible and ornamental pomegranate against* S. mansoni* (Egyptian strain) were evaluated* in vitro* and in experimentally infected mice. This study was the first one investigating the efficacy of ornamental pomegranate against* S. mansoni*. Moreover, as a first step,* in vitro* antischistosomal studies were performed on adult worms.

In the present study, the* in vitro* study established the antischistosomal activity of LEP, SEP, LOP, and SOP extracts on* S. mansoni* adult worms concerning (mating, motility, survival time, and tegumental alterations) the worms at different concentrations (100, 300, and 500 *μ*g/mL). The observed effects were dose-dependent, with 500 *μ*g/mL being the most effective one in a shorter period of incubation. All the tested extracts caused unpairing of couple worms, slow contractions, motility reduction, and paralysis causing the parasites death most of the time. Noel, in his study, explained that paralysis was associated with important neurotransmitters or neuromodulators such as dopamine, acetylcholine, and/or serotonin [39].

SEP and LEP extracts were more efficient mating inhibitors than SOP and LOP extracts. SEP extract (500 mg/kg) was as effective as PZQ* in vitro*. Earlier studies by Pica-Mattoccia and Cioli [40] informed about the PZQ effects on the worms, causing contractions whenever the parasite was exposed to concentrations 0.1 and 1 *μ*g/mL. However, it was known that PZQ caused a quick calcium influx followed by contraction, paralysis, and tegument destruction.

The use of inverted optical microscopy did not allow detailing the tegumental changes presented in the parasite; a qualitative analysis to evaluate the tegumental damage of specimens after treatment* in vitro* through the scanning electron microscopy (SEM) was used in this study. SEM had been employed by several authors in order to elucidate the mechanisms of action of drugs/compounds used in the experimental treatment of schistosomiasis [9, 31, 41].

The changes induced by treatments with ethanolic extracts of dried edible and ornamental pomegranate were related to damage in suckers, oral and acetabular in both male and female schistosomes. SEM examinations of adult schistosomes showed that the treatments caused an extensive peeling of the integument especially in the dorsal region, resulting in the exposure of the antigens of this surface. Furthermore, blebs were visible on the tegument of male worms exposed to treatment with pomegranate extracts. Similar results were observed by de Oliveira et al. [9] who evaluated the* in vitro* effect of crude dichloromethane and aqueous fraction extracts of* Baccharis trimera* on* S. mansoni*. It is believed that the morphological changes caused by a drug/compound with schistosomicidal activity over sarcoplasmic membrane and the tegument of the parasite may be accompanied by an increasing of the exposure to antigens on the surface of the worm. These changes were identified and connected with the host immune-response, required to complement the activity of the drug. For this reason, the tegument of schistosomes had been investigated in the development of new antischistosomal drugs since the late 40s to the present days [9, 42].

In the present study, the* in vivo* antischistosomal activity of LEP, SEP, LOP, and SOP extracts on* S. mansoni* infected mice was evaluated concerning histopathological changes (hepatic inflammation, schistosomal granulomas affection, and number of eggs in liver tissues) and immunological responses through determination of iNOS reactivity. The tested extracts showed dose-dependent reduction in both granuloma diameter and number, number of eggs in liver tissues, liver inflammatory infiltration, and fibrosis compared to infected untreated control groups. SEP and LEP extracts were more effective than SOP in reducing granuloma number and diameter. SEP extract showed significant reduction in inflammatory liver infiltration and hepatic fibrosis similar to PZQ. LEP showed moderate effect, while SOP and LOP extracts showed less prominent effects. The stem bark extracts (SEP and SOP) were almost better than their corresponding leaf extracts (LEP and LOP). SEP extract at the higher dose showed significant results compared to PZQ in reducing granuloma number and diameter. This reduction in the size of granulomatous inflammation indicated an anti-inflammatory effect of the used extracts. Concerning bilharzial eggs in tissues of infected animals, there was variable response with different extracts, where LOP and SOP extracts reduced the number of eggs in the liver tissues at the higher dose, while no eggs were found in liver tissues with LEP at the same dose and SEP extracts at the lower dose compared to PZQ. Comparable results were obtained by previous trials* in vitro* and* in vivo* conducted by the other authors, using compounds isolated from* Piper tuberculatum*, 8-hydroxyquinoline derivatives from* Artemisia annua,* and* Baccharis trimera* that have demonstrated activity against* S. mansoni* [8, 9, 43].

Nitric oxide (NO) is an endogenously secreted free radical, formed as a byproduct of conversion of arginine and oxygen into citrulline in an enzymatic reaction mediated by NO synthase (NOS). Three NOS isoforms have been described to date, inducible NOS (iNOS), which is expressed in response to proinflammatory cytokines. NO production is upregulated in response to parasitic infection. During infection with* S. mansoni*, there is prolonged production of large amounts of NO, so hepatic iNOS is upregulated in* Schistosoma* infected mice, indicating that NO production is a part of an innate immune-response [44, 45]. In our study, iNOS expression was measured by immunohistochemical methods either in hepatocytes or in inflammatory cells. For cytoplasmic iNOS expression in hepatocytes, it decreased with SEP, LEP, and LOP extracts compared to high expression rate in infected untreated control group, while SOP extract failed to reduce iNOS expression. SEP extract produced the highest decrease in iNOS expression at both doses used compared to PZQ group, while LEP and LOP extracts caused weak expression of iNOs at only the higher dose, so the inhibitory effect of the extracts on iNOs expression was dose-dependent. Regarding iNOs expression in inflammatory cells, only SEP and LEP extracts showed inhibitory effect, where SEP abolished iNOS expression at both doses used which was comparable to PZQ group, while LEP at the higher dose caused weak expression of iNOs.

Pomegranate is of a great interest to research in pharmaceutical and new drug development fields because of its distinctive bioactivities, such as hypolipidemic, antiviral, antifungal, antineoplastic, anti-inflammatory, antimutagenic, antioxidant, antibacterial, and antidiarrheal [46–49]. Few studies investigated the effects of edible pomegranate as antischistosomal alternative and reported similar changes in the motility and in the survival rates of the parasite [12, 18, 19]

By reviewing all the available literature, no previous works came across on the use of any ornamental pomegranate extract against* S. mansoni*. Hence, the present work was the first one to prove its antischistosomal activities.

The pharmacological properties of various different parts of this plant have been attributed to its high content of bioactive secondary metabolites, such as polyphenols glycosides, triterpenes, sterols, flavonoids, anthocyanins, triglycerides, tannins, and alkaloids [20, 50].

Pomegranate and its constituents have safely been consumed for centuries without adverse effects. Studies of pomegranate constituents in animals at concentrations and levels commonly used in folk and traditional medicine did not report any toxic effects [51].

## 6. Conclusion

Ornamental and edible pomegranate extracts have* in vitro* and* in vivo* antischistosomal activity against* S. mansoni*. The* in vitro* activity was manifested in couple worm's separation and reduction or complete loss of motor activity and lethality and ultramorphological changes in adult worms. The* in vivo* activity was manifested in reduction of hepatic granulomas number and diameter, decrease of number of bilharzial eggs in liver tissues, less liver inflammatory infiltration, less hepatic fibrosis, and decreased iNOS expression, thus indicating anti-inflammatory effect. Extracts of edible pomegranate were more effective than those of ornamental pomegranate. The highest antischistosomal activity was observed for the ethanolic stem bark extract of edible pomegranate, which gave comparable results to PZQ both* in vitro* and* in vivo*. More studies are needed in order to isolate and identify pomegranate active compounds against the worm and to understand pomegranate mechanism of action on the tegument.

## Figures and Tables

**Table 1:** *In vitro* effects of leaves and stem bark ethanolic extracts of edible and ornamental pomegranate on adult worms of *S. mansoni* after 12 h incubation period.

Group	Conc (*µ*g/mL)	Incubation period (h)	Number of separated worms (%)	% of worm mortality	% of worms with tegumental alteration	
					Partial	Extensive
SEP	500	2	95	90	—	—
		4	99	100	10	90
		6	100	100	—	—
		12	100	100	—	—
	300	2	87	80	—	—
		4	99	99	—	—
		6	99	100	30	70
		12	100	100	—	—
	100	2	71	60	—	—
		4	80	65	—	—
		6	95	90	—	—
		12	100	100	50	50

LEP	500	2	80	0	—	—
		4	85	45	—	—
		6	99	100	20	80
		12	100	100	—	—
	300	2	75	0	—	—
		4	85	70	—	—
		6	99	90	—	—
		12	100	100	15	85
	100	2	65	0	—	—
		4	80	0	—	—
		6	95	90	—	—
		12	100	100	60	40

LOP	500	2	40	0	—	—
		4	75	25	—	—
		6	85	100	20	80
		12	100	100	—	—
	300	2	30	0	—	—
		4	65	12	—	—
		6	75	34	—	—
		12	100	100	50	50
	100	2	20	0	—	—
		4	55	0	—	—
		6	70	0	—	—
		12	100	98	70	30

SOP	500	2	20	10	—	—
		4	60	45	—	—
		6	76	100	30	70
		12	100	100	—	—
	300	2	18	0	—	—
		4	60	0	—	—
		6	70	90	—	—
		12	100	100	85	15
	100	2	15	0	—	—
		4	55	0	—	—
		6	70	90	—	—
		12	100	100	90	10

PZQ	10	2	95	95	—	—
		4	100	100	25	75

Control −ve		2	0	0	—	—
		4	60	0	—	—
		6	70	10	—	—
		12	100	100	0	0

Incubation period: 12 h; control –ve in RPMI-1640 and medium with 3% tween 80 in 0.9% saline.

**Table 2:** Effect of oral administration of different doses of SEP, LEP, LOP, and SOP extracts *in vivo*.

Group	Dose (mg/kg)	Granuloma number		Granuloma diameter (GD)		Eggs in liver tissue	Immunohistochemical findings	
		Mean ± SE	Reduction%	Mean (*μ*m) ± SE	Reduction%		iNos hepatocytes	iNos inflammatory cells
SEP (G5&G7)	600	51.2^*∗∗*^	57.8	141.2 ± 22.1^*∗∗*^	40.1	Absent	Weakest	Negative
	800	45.3^*∗∗*^	62.7	139.2 ± 24.5^*∗∗*^	40.9	Absent	Weakest	Negative

LEP (G4&G6)	600	59.1^*∗*^	51.3	154.3 ± 11.2^*∗∗*^	34.5	Numerous	Strong	Strong
	800	55.7^*∗∗*^	54.1	148.1 ± 22.1^*∗∗*^	37.2	Absent	Weak	Weak

LOP (G8&G10)	600	63.2^*∗*^	47.9	168.1 ± 22.1^*∗*^	28.7	Numerous	Strong	Strong
	800	60.8^*∗*^	49.9	164.5 ± 21.2	30.2	Few	Weak	Strong

SOP (9&G11)	600	68.2^*∗*^	43.8	189.4 ± 24.5^*∗*^	19.6	Numerous	Strong	Strong
	800	57.3^*∗∗*^	52.8	176.2 ± 25.1^*∗*^	25.2	Few	Strong	Strong

PZQ(G3)	200	39.4^*∗*^	67.6	135.4 ± 20.3	42.5	Absent	Weakest	Negative

Infected untreated controls (G1&G2)		121.3	—	235.7 ± 16.1	—	Numerous	Strong	Strong

The difference was significant at ^*∗*^ *P* < 0.01 and ^*∗∗*^ *P* < 0.001 compared to infected untreated control group.
